# Intestinal ischemia after cardiac surgery: analysis of a large registry

**DOI:** 10.1186/1749-8090-8-156

**Published:** 2013-06-18

**Authors:** Johan Nilsson, Erika Hansson, Bodil Andersson

**Affiliations:** 1 Department of Cardiothoracic Surgery, Clinical Sciences, Lund University and Skane University Hospital, Lund, Sweden; 2 Department of Surgery, Clinical Sciences, Lund University and Skane University Hospital, Lund, Sweden

**Keywords:** Cardiac surgery, Gastrointestinal complications, Intestinal ischemia, Outcome, Risk factors

## Abstract

**Background:**

Intestinal ischemia after cardiac surgery is a rare but severe complication with a high mortality. Early surgery can be lifesaving. The aim was to analyze the incidence, outcome, and risk factors for these patients.

**Methods:**

A prospectively collected database with patients who underwent 18,879 cardiac surgical procedures between 1996 and 2011 was investigated. All patients with registered gastrointestinal complications were retrospectively reviewed. Univariate and multivariate analyses were performed to compare patients with and without intestinal ischemia.

**Results:**

Seventeen patients suffered from intestinal ischemia (0.09%), 10 of whom (59%) died. By investigating preoperative parameters independent risk factors were steroids, peripheral vascular disease, cardiogenic shock, and New York Heart Association class 4. When including pre-, per-, and postoperative parameters, only postoperative ones were significant, including elevated creatinine (> 200 μmol/L), prolonged ventilator time, need for intra-aortic balloon pump, and cerebrovascular insult (CVI). The gastrointestinal complications score (GICS) showed a ROC area of 0.87. This was superior compared with EuroSCORE (0.74), to predict intestinal ischemia.

**Conclusions:**

Intestinal ischemia after cardiac surgery is more common in patients with a poor cardiac state, but the use of steroids, peripheral vascular disease, postoperative kidney failure, and CVI were also predictive. GICS score, developed for all GI complications after cardiac surgery, is also of value in predicting this particular complication. The risk factors presented can be used as an aid in the diagnosis of these patients.

## Background

Gastrointestinal (GI) emergencies are infrequent but severe complications after cardiac surgery procedures, with an incidence of 0.4-2.9% [1,2]. The group is heterogeneous, including diagnoses such as upper and lower GI bleeding, acute pancreatitis, acute cholecystitis, perforation of the GI tract, paralytic ileus, and visceral ischemia. Intestinal ischemia is one of the most severe complications, with a mortality rate of 46-100% [3,4].

It can be a challenge to reach an early diagnosis. Patients with GI complications frequently present with atypical symptoms, often have several underlying diseases, and drug therapies, and may be unable to describe symptoms or react to examination due to sedation and analgesia. With intestinal ischemia, a delayed diagnosis and surgical intervention can be fatal [5]. Various demographic and surgical variables and postoperative events have been suggested as risk factors for GI complications [1,6-9], and also for intestinal ischemia in particular [5,10-12]. This identification is important and can lead to earlier diagnosis and treatment.

Intestinal ischemia after cardiac surgery most often is due to a non-occlusive mesenteric ischemia (NOMI) [13,14]. This condition was first described in 1958, and even though the exact pathophysiological mechanism is not understood, it is related to a reduction in the splanchnic blood flow, which can be due to low cardiac output, and it may also be aggravated by inotropic support such as vasopressors, and by pre-existing atherosclerosis [13]. The ischemia is more seldom a cause of arterial emboli or thrombosis and venous thromboembolism [4].

The aim of this study was to investigate the incidence of intestinal ischemia after cardiac surgery over time, and to identify risk factors for the disease and patient outcome by using a large database with prospectively collected material. We compared patients with suspected ischemia who underwent a negative laparotomy and patients diagnosed with intestinal ischemia.

## Methods

Between January 1996 and December 2011, data from adult patients who underwent a total of 19,677 cardiac surgery procedures at Skane University Hospital were registered prospectively. We excluded patients who had coronary artery bypass grafting (CABG) without extracorporeal circulation (*N* = 452), surgery due to dissection or aneurysm in the aorta descendens (*N* = 87), heart transplantation (*N* = 205), laser surgery (*N* = 32), or were < 18 years old (*N* = 21). The remaining 18,590 patients, who had 18,879 operations, were included for further analysis. CABG (*N* = 12,658) was the most common procedure, followed by various heart valve procedures (*N* = 2,220), CABG and heart valve procedures combined (*N* = 2,055) and miscellaneous procedures, e.g. post-infarction septal rupture, aortic dissection type A, and ascending aortic aneurysm, (*N* = 1,946).

The cardiac surgery database contains a total of 248 variables including preoperative, peroperative, and postoperative parameters. Variables previously described in the literature and risk factors from the European system for cardiac operative risk evaluation (EuroSCORE) [15] were chosen for further analysis. EuroSCORE, Higgins and Parsonnet score, that are general risk stratification systems designed to determine overall in-house or 30-day mortality, was evaluated for their ability to predict intestinal ischemia. A score specific for GI complications (GICS score) was also evaluated [9].

The case records of all patients registered as having had a GI complication were retrospectively reviewed and classified according to Andersson et al. [1]. Patients without GI complications were included in the control group. Patients who underwent a negative laparotomy were included in a separate group.

Prolonged ventilator time was defined as the use of a ventilator for more than 24 h after cardiac surgery. The need for inotropic support was registered if the patient required one or several inotropic drugs, e.g. norepinephrine, dobutamine, and dopamine, for more than 48 h. Postoperative renal failure was defined as a serum creatinine level of above 200 μmol/L. Intestinal ischemia was defined as ischemia diagnosed at endoscopy, abdominal surgery, or autopsy.

### Statistical analysis

Values are given as median and interquartile range for continuous variables. For categorical data, absolute numbers and percentages are given. Univariate analysis for continuous variables was done with the Wilcoxon test. Categorical variables were analyzed by Pearson’s test, except when the expected frequencies were less than 5, in which case the Fisher’s exact test was used. The analysis was based on available data. A probability level of less than 0.05 was considered significant. Multivariate analysis was performed using stepwise logistic regression. The inclusion for the full model was *p* < 0.2. The limit for stepwise backward elimination was *p* < 0.1. The discriminatory power of the different scoring models was evaluated by calculation of the area under the receiver operating characteristic (ROC) curves, with 95% confidence limits. To compare the areas under the resulting ROC curves, the non-parametric approach described by DeLong et al. [16] was used. Missing values were replaced using the probability imputation technique. This was done before the selected variables from the univariate analysis were included in the multiple variable analyses and before the risk score was calculated [17].

Statistical analyses were performed with the Hmisc, Survival and Design packages of R software version 2.15.1, 2012 (R Foundation for Statistical Computing, Vienna, Austria).

The Ethics Committee for Clinical Research at Lund University, Sweden approved the study. Written informed consent was not obtained from the patients for publication of this report or any accompanying images, since we report of a large population and not about an individual patient. No image of an individual patient is accompanied.

## Results

### Characteristics of patients with intestinal ischemia

Seventeen patients, including 5 men and 12 women, were diagnosed as having intestinal ischemia during the study period. Median age was 69 (60–75) years and median body mass index (BMI) was 26 (24–29) kg/m^2^. Ten of the patients (59%) died due to the complication.

### Incidence of intestinal ischemia

Since intestinal ischemia after cardiac surgery is a rare event, even when large databases are investigated, a limited number of cases are identified. The present study present the lowest described incidence of this complication (0.09%) with a decreasing incidence during the study period from 0.61% (*N* = 10) during the first 5 years to 0.06% (*N* = 7) during the last 11 years.

### Endoscopic and radiological examinations and abdominal surgery

In all but one patient, a surgeon was consulted. Seven patients underwent endoscopy (sigmoidoscopy or colonoscopy), which was diagnostic in 6 cases. All of these patients later had an abdominal exploration. Those who did not have an endoscopy either went directly for exploration (*N* = 8) or the ischemia was diagnosed at autopsy (*N* = 2). Abdominal X-ray was the only radiological examination in 5 of the cases with ischemia. All showed signs of paralysis with dilation of the intestines. Computed tomography (CT) was performed in 5 cases. In 2 cases, this showed dilatation of the small bowel or the colon, and in 2 cases also mural enhancement of the caecum and the distal ileum or the small bowel. In one patient, there was sign of pancreatitis with peripancreatic inflammation. In 2 cases, ultrasound was combined with a CT-scan and in one of these an X-ray was also conducted. The cardiac surgery and abdominal surgery performed for this group are presented in Table 1.

**Table 1:** Cardiac surgical procedure and abdominal surgery on patients with intestinal ischemia

**Abdominal operation (number of patients)**	**Cardiac surgical procedure (number of patients)**
Small bowel resection (1)	CABG (1)
Small bowel resection and embolectomy (1)	CABG + mitrale valve surgery (1)
Ileocecal resection (1)	CABG + VSD (1)
Hemicolectomy (1)	CABG (1)
Left sided colectomy and colostomy (1)	CABG (1)
Colectomy, ileostomy (4)	Surgery to aorta ascendens, including one with aortic valve replacement (2), CABG (1), CABG with embolectomy in leg (1)
Hemicolecomy and colectomy with ileostomy, both with cholecystectomy (2)	CABG (2)
Explorative laparotomy - no resection due to massive bowel ischemia (4)	CABG (2), Aortic valve surgery (1), Left ventricular assist device (1)
No surgery* (2)	CABG (2)

*CABG* coronary artery bypass grafting, *VSD* ventricular septal defect, *diagnosis at autopsy.

### Univariate risk factors for intestinal ischemia

General patient characteristics, and preoperative state, and postoperative course are compared between patients with and without intestinal ischemia, operated during the time period (Tables 2 and 3).

**Table 2:** Preoperative univariate risk factors for intestinal ischemia after cardiac surgery

**Variable**	***N***	**Control group *N*** **=** **18862**	**Patients with intestinal ischemia *N*** **=** **17**	***p *value**
Age (years)*	18879	69 (60–75)	69 (59–73)	0.45
Female ge*n*der	18879	5187 (27)	6 (35)	0.47
BMI (kg/m^2^)*	15066	26 (24–29)	25 (22–27)	0.15
Haemoglobin (g/L)*	15859	135 (123–145)	130 (118–140)	0.56
Creatinine > 200 (μmol/L)	18144	340 (2)	2 (12)	0.04
Dialysis	18652	226 (1)	0	1
Hemodynamic instable	18876	272 (1)	2 (12)	<0.001
Smoking	8518	849 (10)	2 (25)	0.19
Emergency cardiac surgery	18879	1923 (10)	7 (41)	<0.001
Chronic obstructive pulmonary disease	18653	1756 (9)	2 (12)	0.67
Stroke	18653	830 (4)	3 (18)	0.038
Peripheral vascular disease	18653	2388 (13)	7 (41)	<0.001
Left ventricular ejection fraction <30 %	18879	1482 (8)	2 (12)	0.39
Instable angina	18879	1542 (8)	5 (29)	0.01
Cardiogenic shock	16091	282 (2)	3 (23)	0.001
Atrial fibrillation	14786	1552 (11)	2 (14)	0.65
Anticoagulants	18879	2640 (14)	5 (29)	0.078
Steroids	18879	212 (1)	2 (12)	0.016
IABP	18871	576 (3)	2 (12)	0.094
Diabetes mellitus	18652	3462 (19)	3 (18)	1
NYHA 4	18879	2652 (12)	9 (53)	<0.001

*N* is the number of non missing values, values in parentheses are percentages, except median (interquartile range)*, *BMI* body mass index, *IABP* intra-aortic balloon pump, *NYHA* New York Heart Association.

**Table 3:** **Per**- **and postoperative univariate risk factors for intestinal ischemia after cardiac surgery**

**Variable**	***N***	**Control group *N*** **=** **18862**	**Patients with intestinal ischemia *N*** **=** **17**	***p *value**
CPB time (minutes)*	18879	92 (71–123)	120 (82–169)	0.026
Creatinine >200 (μmol/L)	16285	598 (4)	10 (59)	<0.001
Dialysis	16361	193 (1)	3 (18)	0.001
Reoperation due to bleeding	18875	942 (5)	3 (18)	0.05
Prolonged ventilator time**	17153	1921 (11)	13 (76)	<0.001
Arrhythmia	14335	4929 (34)	8 (62)	0.074
Atrial fibrillation	16568	4998 (30)	8 (47)	0.18
IABP	16284	282 (2)	4 (24)	<0.001
Intropine >24 h	16284	1039 (6)	10 (59)	<0.001
Cardiac infarction	16284	405 (2)	4 (24)	<0.001
Cerebrovascular insult	16284	173 (1)	4 (24)	<0.001

*N* is the number of non missing values, values in parentheses are percentages, except median (interquartile range)*, *CPB* cardiopulmonary bypass time, *IABP* intra-aortic balloon pump, ** 24 hours after cardiac surgery.

**Figure 1:**
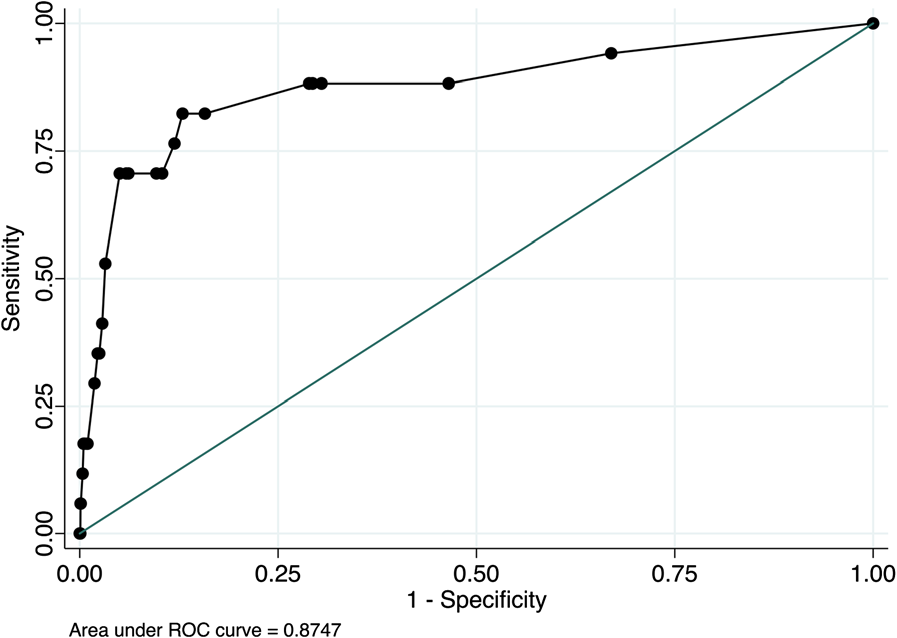
ROC area for GICS score in predicting intestinal ischemia after cardiac surgery.

The well-known cardiac surgery risk scores EuroSCORE, Parsonnet and Higgins score, and also the fairly new GICS score [9], were compared between patient groups (Table 4). The discrimination, represented by the area under the receiver operating characteristic (ROC) curve, was 0.87 (95% CI 0.77-0.98) for the GICS score (Figure 1). For EuroSCORE, the ROC area was 0.74 (95% CI 0.61-0.86).

**Table 4:** Cardiac surgery scoring systems as predictors for intestinal ischemia

**Scoring system**	**Control group (*N*** **=** **18862)**	**Patients with intestinal ischemia (*N*** **=** **17)**	***p *value**
Euro SCORE	5.0 (3.0-8.0)	8.0 (7.0-12)	<0.001
Higgins score	2.0 (1.0-5.0)	7.0 (4.0-10)	<0.001
Parsonnet score	9.0 (3.0-16)	10 (5.0-17)	0.81
GICS score	2.0 (0.0-4.5)	9.0 (6.0-11)	<0.001

### Multivariate risk factors for intestinal ischemia

Data from the univariate analysis with *p* < 0.200 and postoperative factors that were unlikely to be secondary to GI complications (e.g. length of stay at the intensive care unit and multiple organ failure) were included in the multivariate model, as presented in Table 5. We also performed a sub analysis including only preoperative factors (Table 6).

**Table 5:** Multivariate risk factors for intestinal ischemia after cardiac surgery

**Variable**	**Odds Ratio (95% confidence interval)**	***p *value**
Postoperative creatinine > 200 (μmol/L)	17.5 (5.8-53)	<0.001
IABP	3.5 (1.0-12)	0.046
Prolonged ventilator time*	6.2 (1.7-23)	0.006
Cerebrovascular insult	7.8 (2.3-27)	0.001

*IABP* intra-aortic balloon pump, *24 hours after cardiac surgery.

**Table 6:** Preoperative multivariate risk factors for intestinal ischemia after cardiac surgery

**Variable**	**Odds Ratio (95% confidence interval)**	***p *value**
Steroids	9.4 (2.1-43)	0.004
Peripheral vascular disease	3.7 (1.4-9.9)	0.008
Cardiogenic chock	4.9 (1.2-19)	0.023
NYHA 4	4.2 (1.6-13)	0.004

*NYHA* New York Heart Association.

### Comparison of patients with intestinal ischemia and patients with negative laparotomy

Four patients, with a median age of 72 (65–77) years, were suspected of having intestinal ischemia but underwent laparotomies with normal findings during the same time period. In both groups, cardiac surgery reoperation was common (2/4 and 9/17) and each of postoperative sepsis, multiple organ failure, kidney failure, and atrial fibrillation was seen in 25-50% in both groups. The time to abdominal surgery was generally longer for the patients with negative laparotomy, 24 (24–39) h as opposed to 8 (8–43) h for patients with intestinal ischemia, but the difference did not reach statistical significance in this small material. Leucocyte count was significantly higher for patients with intestinal ischemia (14 (10–23) 10^9^/L as opposed to 8.2 (7.5-9.1) 10^9^/L for patients with negative laparotomy; *p* = 0.018), but no other laboratory parameters showed any significant difference, including lactate (3.0 (2.1-6.3) mmol/L and 3.0 (2.1-6.3) mmol/L, respectively; *p* = 0.91).

## Discussion

In this single-center study, based on a large prospectively collected database, intestinal ischemia after cardiac surgery was investigated. We found a lower incidence, 0.09%, than previously presented, but the mortality rate was comparable with others [4,10,12,18-20]. Risk factors were identified that can aid in the diagnosis. To reduce the delay in diagnosis and allow effective use of all therapeutic options, a high index of suspicion for intestinal ischemia after cardiac surgery is warranted, in order to reduce mortality.

Patients with intestinal ischemia after cardiac surgery often have vague and non-specific symptoms. They are often ventilated and sedated, making them unable to react to physical examination. The difficulty in making the diagnosis contributes to the dismal outcome, despite advances in critical care. Thus, in a patient with a septic condition early after surgery, this diagnosis must always be considered. In the present study, when investigating preoperative factors we found the use of steroids, peripheral vascular disease, cardiogenic chock, and NYHA class 4 to be independent prognostic factors for development of the complication. Peripheral vascular disease has previously been pointed out as a risk factor as well as a poor preoperative cardiac condition [5,10,12]. In one older publication, the use of steroids - but in that case postoperatively - was a factor associated with an increased risk of general surgical complications after cardiac surgery [21]. In our study, advanced age, previously suggested to be associated with a higher risk [4,10,18] was not found to be a risk factor.

When investigating relevant factors from the entire hospital stay, we found only postoperative factors, including elevated creatinine, the need for IABP, prolonged ventilator time, and CVI, to be independent risk factors. The need for IABP (a commonly used form of circulatory support for patients with postoperative low cardiac output syndrome) has previously been noted [5,12], as has prolonged ventilation and dialysis support [10]. Stroke has been found to be more common in patients with gastrointestinal complications in cardiac surgery, but previously not described as an independent risk factor [22].

The GICS score [9], which was developed as a scoring model for all GI complications after cardiac surgery, was also predictive for intestinal ischemia alone, with a ROC area of 0.87. This model includes the factors age > 80 years, active smoker, preoperative inotropic support, NYHA class 3 and 4, cardiopulmonary bypass time of > 150 min, and the postoperative factors atrial fibrillation, heart failure, vascular complication, and reoperation due to bleeding.

The most common pathophysiological explanation for intestinal ischemia in these patients is the systemic hypo perfusion state and splanchnic vasoconstriction that lead to NOMI [13,14]. In some studies, survivors of this complication have had surgical intervention earlier [5,11]. However, early laparotomies do not necessarily mean survival in cases of extensive ischemia [23]. An alternative to laparotomy would be diagnostic laparoscopy [24]. Angiographically proven NOMI can also be treated with selective intra-arterial bolus injection and subsequent intra-arterial infusion of e.g. tolazoline, papaverine, or prostaglandin E2 [12,20,25]. In a recently published prospective study of 865 patients arterial angiography was performed if NOMI was suspected, with diagnosis of 78 cases of NOMI and 10 with unremarkable findings [26]. This is, however, a much higher incidence than is seen for clinically relevant mesenteric ischemia.

Four patients in this patient material were subjected to negative laparotomies, and we analyzed differences, including in laboratory findings. No major differences were found between the groups, except that leucocyte count was higher for patients with intestinal ischemia, and the time to abdominal surgery tended to be longer for the patients with a negative laparotomy. Edwards et al. [27] concluded that no clinical, biochemical, or hematological marker has been shown to be discriminatory for ischemia, and plain radiography does not reliably diagnose mesenteric ischemia. Pathological laboratory findings such as elevated lactate are late presentations, as well as findings on CT such as mural enhancement and gas in the bowel wall. Larger studies are currently lacking to reliably advocate the routine clinical use of novel markers such as mucosal damage markers, e.g. intestinal fatty acid-binding protein [28].

It has been suggested that patients should undergo routine endoscopic examination of the colon early after bypass and when clinically indicated [29]. In the present study, almost half of the patients underwent coloscopy, most of them with diagnostic findings. However, both radiological and endoscopic investigations are time consuming. Patients with a high index of suspicion of the diagnosis should therefore be taken directly to intervention.

The incidence of intestinal ischemia after cardiac surgery varies in the literature. The findings of the present study are in line with the observation of Allen et al. [18]. It is worth noting that a decrease in the incidence was seen during our study period. There was no obvious specific change in postoperative care, but regarding preoperative status, fewer patients with one of the identified preoperative risk factors - cardiogenic shock – had been subjected to emergency cardiac surgery during the last years. Instead, these patients were preferentially managed with percutaneous coronary intervention. We believe that this could explain the lower incidence to some extent.

Some limitations that must be mentioned are those inherent in research based on large databases, including missing values and also a lack of warranted parameters, e.g. laboratory data. Since the diagnosis is infrequent, the numbers of patients with the complication are limited, which could mean that there is a risk of failing to identify parameters of importance.

## Conclusions

We found intestinal ischemia after cardiac surgery to be an uncommon complication, but associated with a high mortality. In our struggle to find characteristics of patients at risk, we identified several risk factors, reflecting patients with a poor preoperative state and postoperative complications, including kidney failure, CVI, prolonged ventilator time, and the use of IABP. In future, we must continue our efforts to diagnose these patients earlier, to reduce mortality. The GICS score has proved to be a useful tool in this important task.

## Abbreviations

BMI: Body mass index; CABG: Coronary artery bypass grafting; CT: Computed tomography; CVI: Cerebrovascular insult; GI: Gastrointestinal; GICS: Gastrointestinal complications score; IABP: Intra-aortic balloon pump; NOMI: Non-occlusive mesenteric ischemia; NYHA: New York Heart Association; ROC: The receiver operating characteristic.

## Competing interests

The authors declare that they have no competing interests.

## Authors’ contributions

JN participated in the design of the study and in data collection, and performed the statistical analysis. EH participated in data collection and drafted the manuscript. BA designed the study, participated in data collection, and wrote the manuscript. All authors read and approved the final manuscript.
